# Efficacy and safety of tirzepatide, dual GLP-1/GIP receptor agonists, in the management of type 2 diabetes: a systematic review and meta-analysis of randomized controlled trials

**DOI:** 10.1186/s13098-023-01198-4

**Published:** 2023-10-30

**Authors:** Qian Zhou, Xingxing Lei, Shunlian Fu, Pan Liu, Cong Long, Yanmei Wang, Zinan Li, Qian Xie, Qiu Chen

**Affiliations:** 1 Hospital of Chengdu University of Traditional Chinese Medicine, Chengdu, 610072 Sichuan China; 2 Present Address: Chengdu, China; 3 Ya’an Polytechnic College Affiliated Hospital, Ya’an, China; 4 Sichuan Integrative Medicine Hospital, chengdu, China; 5 Hospital of Chengdu University of Traditional Chinese Medicine, No. 39, Shi-er-Qiao Road, Chengdu, 610072 Sichuan Province People’s Republic of China

**Keywords:** GLP-1 receptor agonists, Dual GIP/GLP-1 receptor agonist, Tirzepatide, Type 2 diabetes, Meta-analysis

## Abstract

**Background:**

Glucose-dependent insulinotropic polypeptide (GIP) and GLP-1 are the main incretin hormones, and be responsible for the insulinotropic incretin effect. The addition of a GIP agonist to a GLP-1agonist has been hypothesized to significantly potentiate the weight-losing and glycemia control effect, which might offer a new therapeutic option in the treatment of type 2 diabetes. The current meta-analysis aims to synthesize evidence of primary efficacy and safety outcomes through clinically randomized controlled trials to evaluate integrated potency and signaling properties.

**Method:**

We conducted comprehensive literature searches in Cochrane Library, Web of Science, Embase and PubMed for relevant literatures investigating the efficacy and/or safety of Tirzepatide published in the English as of May 30, 2023 was retrieved. We synthesized results using standardized mean differences (SMDs) and 95% confidence intervals (95 CIs) for continuous outcomes, and odds ratios (ORs) along with 95 Cis for dichotomous outcomes. All analyses were done using Revman version 5.3, STATA version 15.1 and the statistical package ‘meta’.

**Results:**

Participants treated with weekly Tirzepatide achieved HbA1c and body weight target values significantly lower than any other comparator without clinically significant increase in the incidence of hypoglycemic events, serious and all-cause fatal adverse events. However, gastrointestinal adverse events and decreased appetite events were reported more frequently with Tirzepatide treatment than with placebo/controls.

**Conclusion:**

The Tirzepatide, a dual GIP/GLP-1 receptor co-agonist, for diabetes therapy has opened a new era on personalized glycemia control and weight loss in a safe manner with broad and promising clinical implications.

**Supplementary Information:**

The online version contains supplementary material available at 10.1186/s13098-023-01198-4.

## Introduction

Type 2 diabetes (T2D) is a chronic metabolic condition marked by hyperglycaemia that requires stepwise addition of multiple glucose-lowering medications as the disease progression [1, 2]. The net result is a viscous cycle of hyperglycaemia leading to continuous deterioration of metabolic function and necessitating insulin therapy in many cases. Obesity is one of the major modifiable risk factors for type 2 diabetes. The parallel rising prevalence of obesity and T2D (name "diabesity") present a principal global health challenge with increased risk for overall mortality [3]. In patients with T2D, Glucagon-like peptide-1 receptor agonists (GLP-1Ras) improve the regulation of glucose homeostasis, weight-losing, and long-term benefit cardiovascular outcomes, which have been demonstrated to be accompanied by improved micro- and macrovascular risk factors [4, 5]. While real-world evidences of the broad metabolic benefits of GLP-1Ra have emerged, many patients do not achieve their individualised glycemic and body weight (BW) targets with the currently approved incretin, making continuous optimization of these agents an important clinical goal [6]. Individualizing the glycemic and BW targets for diabesity patients is now the guideline-recommended strategy [7], how therapy could accomplish this is unknown. Moreover, dose dependent gastrointestinal effects of GLP-1Ra limits the efficacy. Therefore, agents possessing GLP-1 pharmacology that can active alternative pathways might expand the therapeutic index for T2D.

Glucose-dependent insulinotropic polypeptide (GIP) and GLP-1 are the main incretin hormones, potentiate glucose-induced insulin secretion and therefore be responsible for the insulinotropic incretin effect [8]. The incretin effect accounts for at least 50% of total insulin secreted after oral glucose consumption [9]. GLP-1 has been exhibited central inhibitory actions on appetite and food intake, comparatively little is seen with the central activity of GIP on appetite [10]. Emerging evidence has illustrated that combining the GLP-1Ras with GIP Ras is an integrated potency to achieve significantly weight-losing along with glycemia control effect [11], which may provide a novel therapeutic option for the treatment of T2D [12]. Tirzepatide (LY3298176), a dual GIP and GLP-1 Ra, was discovered by engineering GLP-1 activity into the GIP sequence, which has the potential to be one of the most effective therapeutics for treating T2D with respect to both glycemia and body weight control as the disease progresses [11]. The preliminary clinical study showed Tirzepatide was superior to titrated insulin degludec (ID) with unprecedented efficacy in HbA_1c_ and body weight in T2DM as approximate thirty percent of patients receiving subcutaneous injection of Tirzepatide 15 mg weekly returned normoglycemia (HbA1C < 5.7% per the American Diabetes Association definition) and a quarter of subjects lost more than fifteen percent (− 7·5 to − 12·9 kg for all Tirzepatide doses) of their weight in a 52-week trial [12]. Furthermore, Tirzepatide has be the first dual agonist (GLP-1 and GIP Ra) to be licensed for diabetes therapy.

Hence, we hypothesized Tirzepatide possess a unique profile tailored of pharmacology with the synergetic effect and signaling properties in regulating extensive metabolic control. We aimed to systematically retrieve all available randomised, placebo-controlled trials of Tirzepatide in individuals with T2D to discuss evidence for this hypothesis, synthesize evidence of primary efficacy and safety outcomes, and evaluate integrated potency and signaling properties through a clinically relevant systematic review and meta-analysis. Our meta-analysis results will help clinicians to determine the optimal application of Tirzepatide in clinical practice and optimize diabetes management strategies for individuals with T2D.

## Method

The protocol for this meta-analysis has been registered on Prospero, the international prospective register of systematic reviews, under the identifier CRD42022355940 (https://www.crd.york.ac.uk/prospero/#myprospero). We adhered to the PRISMA (Preferred Reporting Items for Systematic Reviews and Meta-analyses) guidelines for conducting, reporting and updating the systematic review and meta-analysis (Additional file 1: Table S1) [13].

### Search strategy

We conducted a literature search to identify published randomized placebo-controlled trials that tested T2D patients with a weekly subcutaneous injection of a maintenance dose of 5, 10, or 15 mg of Tirzepatide as glucose-lowering medication. Cochrane Library, Web of Science, Embase and PubMed were searched comprehensively for relevant literatures investigating the efficacy and/or safety of Tirzepatide for reports published in the English and up to 30 May 2023. The search strategy included medical subject heading (MeSH) terms and scientific name of the keywords ‘Tirzepatide’, ‘ly3298176’ and ‘Twincretin’. We also manually searched the databases to identify any additional studies, reviews and references lists of eligible studies and conference proceedings.

### Study selection

This updated review included trials with a cross-over or parallel design that compared Tirzepatide at three doses (5 mg, 10 mg and 15 mg) with placebo or various hypoglycemic comparators in patients with T2D and evaluate any predefined outcomes of interest, efficacy and safety parameters related to the treatment. The criteria of the individuals with T2D meet the following requirements: (a) individuals with a medical history of physician-diagnosed T2D; or (b) the individuals without prior history of T2D, but with uncontrolled glycemic after admission or taking medication and a new diagnosis of T2D comprised the diabetic group. Eligible study participants were adults (aged 18 years or older) diagnosed with type 2 diabetes, irrespective of either diet and exercise alone or oral antihyperglycaemic medication, for at least 3 months before screening. The databases search results were imported into the reference management software (Endnote V 9.3) and juxtaposed with the results from other search sources after data deduplication. All citations that were generated by the literature search were screened by one reviewer (QZ) and verified by a second independent reviewer (XX) on the basis of title and abstract. The full-text literatures were obtained for all citations of interest and were assessed were screened independently by two reviewers (CL, QC), and any disagreements were resolved by a third reviewer (SL).

### Data extraction

We used pre designed forms to extract data for eligible studies. Data extraction procedure included bibliographic information, participants’ demographics and clinical characteristics when present, and information on intervention and outcomes. Two reviewers (SF, QC) independently extracted data in duplicate and tabulated all information extracted from the included studies. Any discrepancies shall be resolved by consensus. Among the included literatures, the primary metabolic parameters end point was the mean absolute changes from baseline in hemoglobin A1c (HbA1c, %), fasting serum glucose (FSG, mg/dL), body weight (BW, kg), and triglyceride (TG, mg/dL), HDL cholesterol (HDL-c, mg/dL), and LDL cholesterol (LDL-c, mg/dL) with Tirzepatide at different doses monotherapy or as adjunctive therapy with various stable antihyperglycaemic therapy, diet and exercise alone or oral medication, and/or insulin, in patients with T2D. Secondary efficacy endpoint was the mean changes of safety and tolerability outcomes of Tirzepatide at different doses. For subgroup, we extracted data at the corresponding time point and glucose-lowering agents of placebo/control group to appraise the efficacy and safety outcomes of Tirzepatide at different doses. Primary outcomes are extracted based on means, standard deviations (or standard error), and the number of patients randomized in each study arm for continuous outcomes. With regard to safety and tolerability outcomes, we considered all adverse events (AEs), serious AEs, Fatal AEs, hypoglycemic events (HEs) (blood glucose level ≤ 5.4 mmol/l), gastrointestinal events (mainly including nausea, vomiting and diarrhoea), decreased appetite, and AEs leading to discontinuation of therapy.

### Quality and risk-of-bias assessment

Included trials were assessed for bias with the Cochrane risk-of-bias tool [14]. Two reviewers (QZ, XX) independently assessed the methodological quality and risk of bias for the primary outcomes in duplicate, and if there were any disagreements, a senior reviewer (QC) would arbitrate. The overall risk of bias was judged to be high in the presence of high bias for one or multiple domains raised concerns, low if all key domains were at low risk of bias, and unclear risk of bias if at any domain was unclear. We evaluated the potential for publication bias and investigated the presence of small study effects for the primary outcomes with visual inspection of the funnel plot and Egger's test. A two tailed P value < 0.05 was considered significant.

### Data synthesis

We synthesized results using standardized mean differences (SMDs) and 95% confidence intervals (95 CIs) for continuous outcomes, and odds ratios (ORs) along with 95 CIs for dichotomous outcomes with a fixed-effect model (Mantel–Haenszel approach) or a random-effect model (DerSimonian–Laird method) based on I^2^ value (I^2^ < 50%, low heterogeneity; I^2^ ≥ 50%, high heterogeneity). For dichotomous outcomes. In the case of missing standard deviations (SDs), we calculated them from standard errors, corresponding 95 CIs, interquartile ranges, or other measures (if available) [15]. All the efficacy estimates were presented as means changes and 95% CIs from baseline unless otherwise noted. We performed separate analysis based on tirzepatide maintenance dose (5 mg, 10 mg or 15 mg) and subsequent subgroup-analyses based on various type of comparators (Placebo, GLP-1 Ra and Insulin). For the potential statistical interstudy heterogeneity, p values were measured from Cochran’s Q test and quantified by using Higgins I^2^ statistic. All analyses were done using ReVman version 5.3, STATA version 15.1 and the statistical package ‘meta’.

## Results

### Study characteristics

**Fig. 1:**
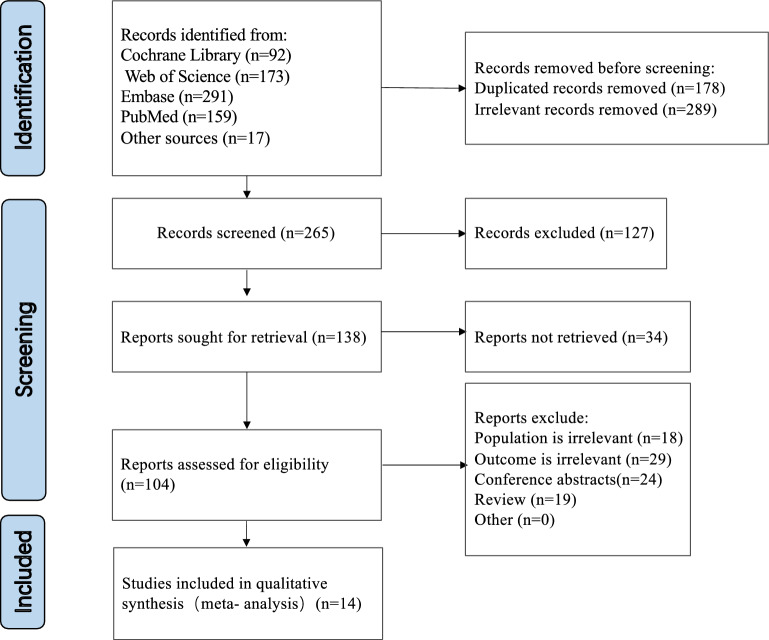
Flowchart of literature search

After detailed screening, altogether 14 trails met the inclusion criteria, the flow chart of the database search and the study selection process was shown in Fig. 1. All trials were of substantial size, with 11,158 patients were eventually included in the meta-analysis (Table 1). These 14 trials include therapy naive patients (only healthy lifestyle education and dietary interventions) and participants receiving background anti-hyperglycaemic therapy comprised metformin, Sodium-glucose cotransporter 2 (SGLT2) inhibitor or insulin, either as monotherapy or in combination with other medications. The comparators included placebo, GLP-1 Ra and insulin. Among them, four studies are placebo controlled, six trials were conducted with GLP-1 Ra as comparators (Dulaglutide and Semaglutide), four trials are long-acting insulin analogues (Insulin Glargine/IG and Insulin Dulaglutide/ ID). The basic characteristics of participants in treatment and placebo/control groups were similar, such as baseline age and diabetes duration, with a weighted means of 57.9 ± 2.6 kg and 8.7 ± 2.4 years, respectively. All trials used a parallel group design, and three were open-label with follow-up duration ranged from 8 to 72 weeks.

**Table 1:** Characteristics of included randomized controlled trials

Author	Year	Country	Study arms	Dose and frequency	Participants	Diabetes duration years	Age (y) Mean ± SD	Duration	BMI
Frías, J. P. [22]	2022	USA	Tirzepatide	5 mg/week	470	9.1 ± 7.16	56.3 ± 10.0	40 weeks	33.8 ± 6.85
				10 mg/week	469	8.4 ± 5.90	57.2 ± 10.5		34.3 ± 6.6
				15 mg/week	470	8.7 ± 6.85	55.9 ± 10.4		34.5 ± 7.11
			Semaglutide	1 mg/week	469	8.3 ± 5.80	56.9 ± 10.8		34.2 ± 7.15
Vadher, K. [20]	2022	UK	Tirzepatide	5 mg/week	470	9.1 ± 7.2	56.3 ± 10.0	40 weeks	33.8 ± 6.9
				10 mg/week	469	8.4 ± 5.9	57.2 ± 10.5		34.3 ± 6.6
				15 mg/week	470	8.7 ± 6.9	55.9 ± 10.4		34.5 ± 7.1
			Semaglutide	2 mg/week	480	8.3 ± 5.8	57.9 ± 10.0		34.2 ± 7.2
Ludvik, B. [16]	2021	Austria	Tirzepatide	5 mg/week	358	NP	57.2 ± 10.1	52 weeks	33.6 ± 5.9
				10 mg/week	360		57.4 ± 9.7		33.4 ± 6.2
				15 mg/week	358		57.5 ± 10.2		33.7 ± 6.1
			Insulin degludec	10 U/day	359		57.5 ± 10.1		33.4 ± 6.1
Frias, J. P. [27]	2020	USA	Tirzepatide	5 mg/week	29	10.5 ± 7.90	61.2 ± 7.56	12 weeks	31.1 ± 4.21
				10 mg/week	28	8.2 ± 4.87	55.5 ± 8.54		32.0 ± 5.56
				15 mg/week	28	8.9 ± 6.35	56.6 ± 9.21		32.0 ± 5.19
			Placebo	NP	26	8.8 ± 6.43	56.0 ± 10.13		32.5 ± 5.70
Dahl, D. [18]	2022	Germany	Tirzepatide	5 mg/week	116	14.1 ± 8.1	62 ± 10	40 weeks	33.6 ± 5.9
				10 mg/week	119	12.6 ± 6.2	60 ± 10		33.4 ± 6.2
				15 mg/week	120	13.7 ± 7.5	61 ± 10		33.4 ± 5.9
			Insulin Glargine	> 20 IU/day or > 0.25 IU/kg/d	120	12.9 ± 7.4	60 ± 10		33.2 ± 6.3
Wilson, J. M. [23]	2020	USA	Tirzepatide	5 mg/week	41	8.9 ± 5.7	57.9 ± 8.2	26 weeks	32.9 ± 5.7
				10 mg/week	47	7.9 ± 5.8	56.5 ± 9.9		32.6 ± 5.8
				15 mg/week	43	8.5 ± 6.1	56.0 ± 7.6		32.2 ± 6.2
			Dulaglutide	1.5 mg/week	33	9.3 ± 7.1	58.7 ± 7.8		32.4 ± 5.4
			Placebo	NP	41	8.6 ± 7.0	56.6 ± 8.9		32.4 ± 6.0
Furihata, K. [61]	2022	Japan	Tirzepatide	5 mg/week	11	7.0 ± 5.0	57.5 ± 7.9	8 weeks	26.7 ± 3.3
				10 mg/week	12	8.4 ± 3.8	56.9 ± 9.5		25.5 ± 2.8
				15 mg/week	16	9.1 ± 4.8	57.7 ± 8.0		26.1 ± 3.1
			Placebo	NP	9	9.5 ± 3.4	57.4 ± 11.6		22.6 ± 2.1
Heise, T. [62]	2022	Germany	Tirzepatide	15 mg/week	45	10.24 ± 5.80	61.1 ± 7.1	28 weeks	NP
			Semaglutide	1 mg/week	44	12.73 ± 6.10	63.7 ± 5.9		
			Placebo	NP	28	10.95 ± 6.78	60.4 ± 7.6		
Inagaki, N. [63]	2022	Japan	Tirzepatide	5 mg/week	159	4.5 (2.1–7.5)	56.8 ± 10.1	52 weeks	28.6 ± 5.4
				10 mg/week	158	5.1 (2.2–8.4)	56.2 ± 10.3		28.0 ± 4.1
				15 mg/week	160	5.1 (2.2–8.4)	56.0 ± 10.7		28.1 ± 4.4
			Dulaglutide	0.75 mg/week	159	5.0 (1.9–8.4)	57.5 ± 10.2		27.8 ± 3.7
Frias, J. P. [12]	2018	USA	Tirzepatide	5 mg/week	55	8.9 ± 5.7	57.9 ± 8.2	26 weeks	32.9 ± 5.7
				10 mg/week	51	7.9 ± 5.8	56.5 ± 9.9		32.6 ± 5.8
				15 mg/week	53	8.5 ± 6.1	56.0 ± 7.6		32.2 ± 6.2
			Dulaglutide	1.5 mg/week	54	9.3 ± 7.1	58.7 ± 7.8		32.4 ± 5.4
			Placebo	NP	51	8.6 ± 7.0	56.6 ± 8.9		32.4 ± 6.0
Rosenstock, J. [21]	2021	USA	Tirzepatide	5 mg/week	121	4.6 ± 5.1	54.1 ± 11.9	40 weeks	32.2 ± 7.0
				10 mg/week	121	4.9 ± 5.6	55.8 ± 10.4		32.2 ± 7.6
				15 mg/week	120	4.8 ± 5.0	52.9 ± 12.3		31.5 ± 5.5
			Placebo	NP	113	4.5 ± 5.9	53.6 ± 12.8		31.7 ± 6.1
Del Prato, S. [17]	2021	USA	Tirzepatide	5 mg/week	329	9.8 (6.2–15.3)	62.9 ± 8.6	52 weeks	32.6 ± 6.06
				10 mg/week	328	10.6 (6.5–16.2)	63.7 ± 8.7		32.8 ± 5.51
				15 mg/week	338	10.4 (5.5–15.7)	63.7 ± 8.6		32.5 ± 5.02
			Insulin Glargine	10 U/day	1000	10.7 (6.3–16.5)	63.8 ± 8.5		32.5 ± 5.55
Gao, L. [19]	2023	China	Tirzepatide	5 mg/week	230	7.43 ± 5.93	53.1 ± 11.2	40 weeks	28.1 ± 3.9
				10 mg/week	228	7.9 ± 5.65	53.5 ± 11.1		27.7 ± 3.8
				15 mg/week	229	7.64 ± 5.63	54.3 ± 11.6		27.8 ± 3.8
			Insulin Glargine		220	7.65 ± 5.72	55.6 ± 11.4		28 ± 4.6
Garvey, W. T. [64]	2023	USA	Tirzepatide	5 mg/week	312	8.8 ± 6.9	54.3 ± 10.7	72 weeks	36.0 ± 6.4
				15 mg/week	311	8.0 ± 6.4	53.6 ± 10.6		35.7 ± 6.1
			Placebo	NP	315	8.8 ± 6.2	54.7 ± 10.5		36.6 ± 7.3

*NR* not reported, *USA* United States of America, *UK* United Kingdom

**Fig. 2:**
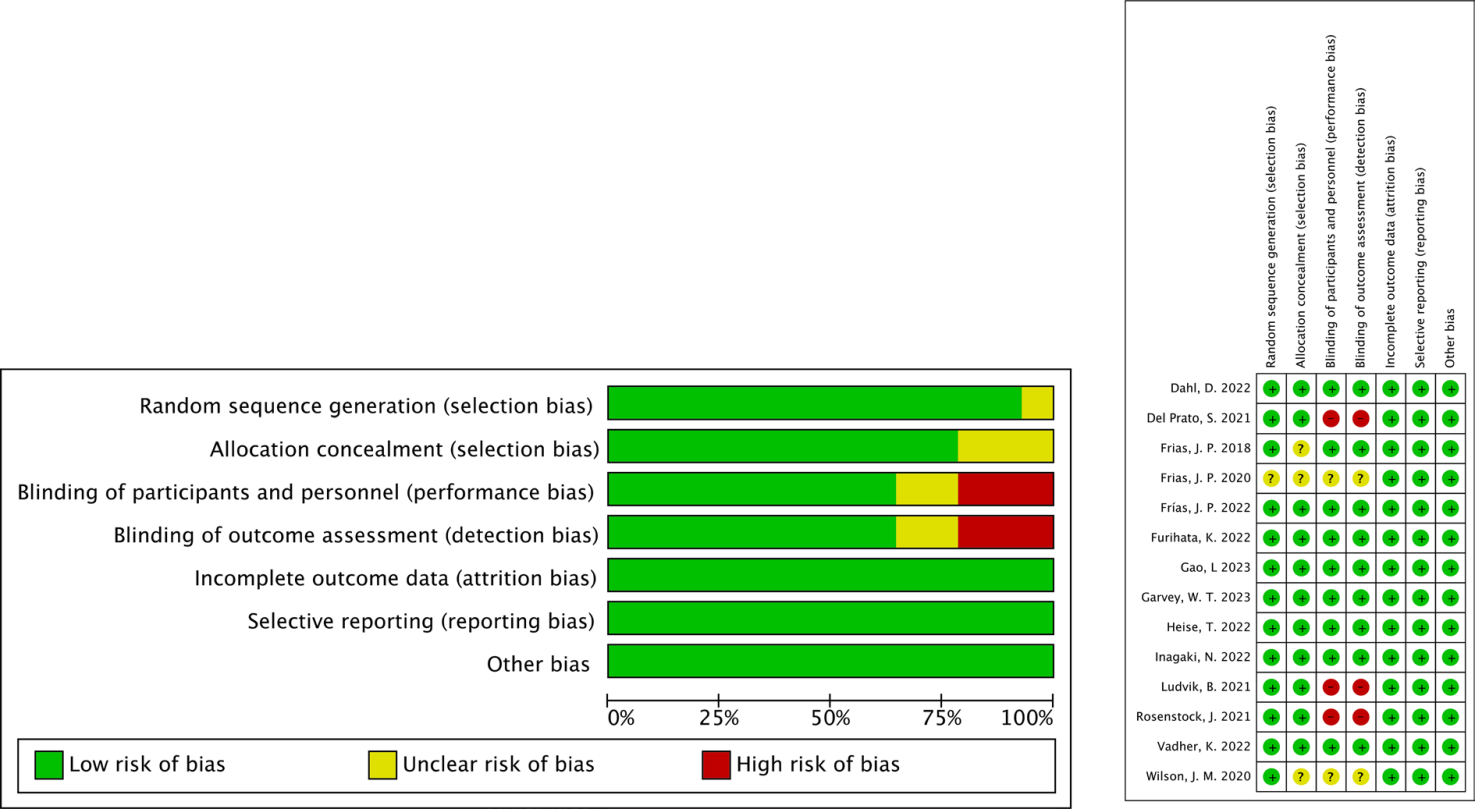
Overall summary of risk of bias in the included studies. + : low risk of bias; −: high risk of bias; ?: unclear risk of bias

**Fig. 3:**
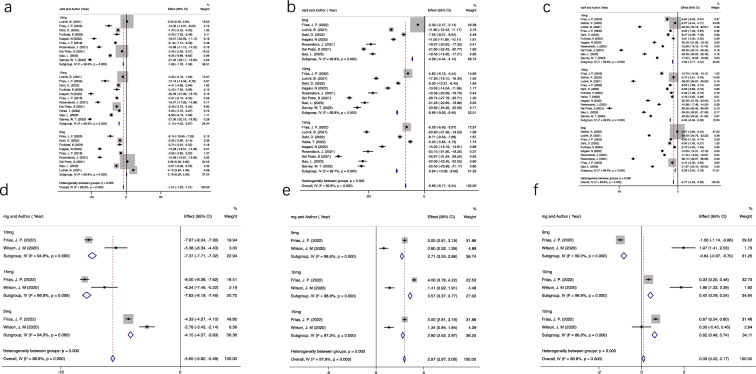
Summary of quantitative data analysis with Random effects or fixed effects SMD (95% CI) estimate with a p-value for analysis of primary efficacy outcomes. ^*^Statistically significant variables at P value < 0.05. **a** Fasting serum glucose, **b** hemoglobin A1c, **c** body weight, **d** triglyceride, **e** HDL cholesterol, **f** LDL cholesterol

All included trials were assessed for bias with the Cochrane risk-of-bias tool and all studies exhibited a low risk of selection bias, performance bias, detection bias, and reporting bias. Therefore, overall quality and risk-of-bias for the primary outcomes were assessed as high quality with a low risk of bias (Figs. 2, 3). Other main baseline characteristics of the included RCTs, such as the design, name and dose of study drugs, study details, demographics and outcome-specific data, are reported in Table 1. In addition, no test for publication bias was conducted due to the limited numbers of the included studies on parameters discussed.

### Glycolipid metabolism

#### Fasting serum glucose

The synthesized results of meta-analyses for available data showed a significant reduction in FSG (SMD = − 1.57, 95% CI: − 1.63 to − 1.51, P < 0.05) versus placebo/controls for combined tirzepatide arms intervention group (Table 2). Regarding the three different doses of the tirzepatide, a significant decrease in FBG of tirzepatide 10 mg (SMD = − 1.68, 95% CI: − 1.78 to − 1.58, P < 0.05), and superior reduction in tirzepatide 15 mg (SMD = − 4.10, 95% CI: − 4.23 to − 3.97, P < 0.05) was observed. However, the opposite results were observed in the tirzepatide 5 mg subgroup (SMD = 0.19, 95% CI: 0.09 to 0.29, P < 0.05). Numbers and types of drug discrepancies, medication histories, experimental design and approaches in detecting might be the source of these inconsistent results. Therefore, we analyzed each efficacy and safety outcome and different doses of tirzepatide separately based on the type of comparators (Placebo, GLP-1 Ra and Insulin).

**Table 2:** Summary of quantitative data analysis with Random effects or fixed effects SMD (95% CI) estimate with a p-value for analysis of primary efficacy outcomes

Primary efficacy outcomes	5 mg	10 mg	15 mg	Overall
FSG	0.19 (0.09, 0.29) P < 0.05	− 1.68 (− 1.78, − 1.58) P < 0.05	− 4.10 (− 4.23, − 3.97) P < 0.05	− 1.57 (− 1.63, − 1.51) P < 0.05
HbA1c	− 4.29 (− 4.44, − 4.14) P < 0.05	− 9.69 (− 9.93, − 9.45) P < 0.05	− 9.84 (− 10.09, − 9.6) P < 0.05	− 6.77 (− 6.77, − 6.54) P < 0.05
BW	− 2.29 (− 2.4, − 2.18) P < 0.05	− 4.56 (− 4.71, − 4.42) P < 0.05	− 6.46 (− 6.64, − 6.28) P < 0.05	− 3.77 (− 3.84, − 3.69) P < 0.05
TG	− 4.153 (− 4.373, − 3.933) P < 0.05	− 7.369 (− 7.714, − 7.023) P < 0.05	− 7.825 (− 8.188, − 7.462) P < 0.05	− 5.651 (− 5.816, − 5.485) P < 0.05
LDL-c	− 0.835 (− 0.967, − 0.703) P < 0.05	0.418 (0.293, 0.543) P < 0.05	0.615 (0.489, 0.741) P < 0.05	0.094 (0.020, 0.167) P < 0.05
HDL-c	2.708 (2.534, 2.881) P < 0.05	3.571 (3.369, 3.774) P < 0.05	2.799 (2.624, 2.974) P < 0.05	2.974 (2.869, 3.079) P < 0.05

*FSG* fasting serum glucose, *HbA1c* hemoglobin A1c, *BW* body weight, *TG* triglyceride, *HDL-c* HDL cholesterol, *LDL-c* LDL cholesterol

*Statistically significant variables at P value < 0.05

Compared with placebo and GLP-1 Ra, all three doses of tirzepatide had a more significant and consistent effect on FSG reduction (all P < 0.05). Compared with insulin (insulin degludec and insulin glargine), except for the 5 mg subgroup (SMD = 0.57, 95% CI: 0.46 to 0.67, P < 0.05). All other subgroups of parameters were consistent with significant statistical significance. Further details can be found in Table 3. There was difference in the glucose lowering effect between insulin and low tirzepatide dose (5 mg). Of note, the mean subcutaneous injection dose of basal insulin during the study duration was 10 U/day with ID in the SURPASS-3 Randomized Clinical Trial [16], 10 U/day with IG in SURPASS-4 Trial [17], > 20 IU/d or > 0.25 IU/kg/d with IG in SURPASS-5 Trial [18], and 0.33 U kg^−1^ d^−1^ with IG in SURPASS-AP-Combo trial [19].

**Table 3:** Summary of quantitative data analysis with Random effects or fixed effects SMD (95% CI) estimate with a p-value for analysis of primary efficacy outcomes based on various type of comparators

Primary efficacy outcomes	Comparator	5 mg	10 mg	15 mg
FSG	GLP-1 RA	− 5.23 (− 5.73, − 4.72) P < 0.05	− 9.73 (− 10.51, − 8.95) P < 0.05	− 6.23 (− 6.74, − 5.71) P < 0.05
	Insulin	0.57 (0.46, 0.67) P < 0.05	− 1.23 (− 1.33, − 1.12) P < 0.05	− 3.6 (− 3.74, − 3.47) P < 0.05
	Placebo	− 9.69 (− 10.59, − 8.79) P < 0.05	− 9.89 (− 10.42, − 9.35) P < 0.05	− 15.84 (− 16.62, − 15.06) P < 0.05
	Overall	0.19 (0.09, 0.29) P < 0.05	− 1.68 (− 1.78, − 1.58) P < 0.05	− 4.1 (4.23, − 3.97) P < 0.05
HbA1c	GLP-1 RA	− 0.93 (− 1.03, − 0.83) P < 0.05	− 1.50 (− 1.62, − 1.39) P < 0.05	− 1.60 (− 1.72, − 1.48) P < 0.05
	Insulin	− 13.3 (− 13.69, − 12.91) P < 0.05	− 17.43 (− 17.93, − 16.93) P < 0.05	− 17.9 (− 18.43, − 17.37) P < 0.05
	Placebo	− 5.54 (− 6.22, − 4.86) P < 0.05	− 10.10 (− 10.73, − 9.47) P < 0.05	− 17.14 (− 18.00, − 16.28) P < 0.05
	Overall	− 1.77 (− 1.87, − 1.68) P < 0.05	− 2.58 (− 2.69, − 2.47) P < 0.05	− 2.62 (− 2.73, − 2.5) P < 0.05
BW	GLP-1 RA	− 5.2 (− 5.45, − 4.96) P < 0.05	− 3.29 (− 3.44, − 3.14) P < 0.05	− 1.83 (− 1.94, − 1.75) P < 0.05
	Insulin	− 7.38 (− 17.86, − 16.90) P < 0.05	− 17.25 (− 17.91, − 16.58) P < 0.05	− 18.51 (− 19.25, − 17.78) P < 0.05
	Placebo	− 12.6 (− 13.12, − 12.09) P < 0.05	− 8.28 (− 9.04, − 7.52) P < 0.05	− 9.55 (− 10.42, − 8.68) P < 0.05
	Overall	− 8.42 (− 8.62, − 8.22) P < 0.05	− 4.14 (− 4.28, − 3.99) P < 0.05	− 2.29 (− 2.39, − 2.18) P < 0.05

*FSG* fasting serum glucose, *HbA1c* hemoglobin A1c, *BW* body weight

*Statistically significant variables at P value < 0.05

#### Glycaemic

All tirzepatide doses (5, 10 and 15 mg) significantly reduced HbA1c percent and were superior to various comparators (Placebo, GLP-1 Ras and Insulin) (Tables 2, 3). The results show evidence of a dose-dependent reduction in HbA1c percent versus placebo with tirzepatide 5 mg (SMD = − 4.29, 95% CI: − 4.44 to − 4.14, P < 0.05), 10 mg (SMD = − 2.306, 95% CI: − 9.93 to − 9.45) and 15 mg (SMD = − 9.84, 95% CI: − 10.09 to − 9.6). In all included 12 literatures, only one article reported that there was no significant difference between Tirzepatide 5 mg and Semaglutide 2 mg in change from baseline in HbA1c at week 40 with an estimated treatment difference (ETD) of 0.07% (95 CI: 0.2–0.34; P = 0.606) [20]. The other 11 articles found that the hypoglycemic hemoglobin effect of three doses (5, 10 and 15 mg) of tirzepatide were superior to any active comparator (placebo, dulaglutide, semaglutide, insulin glargine and insulin degludec). In SURPASS 1–5 Trials, the synthesized results of HbA_1c_ reduction were between 1.69 to 2.58% across the doses ranging from 5 to 15 mg of tirzepatide once a week, with approximately 24–30 weeks of tirzepatide treatment to reach a new plateau of HbA_1c_ and FPG [16–18, 21, 22].

#### Body weight

Similar to the results of HbA1c, a statistical dose-dependent reduction in BW of three tirzepatide dose groups when compared to the placebo group was observed (all P < 0.05, Table 2). Consistently, compared with various anti-hyperglycaemic agents, patients receiving all tirzepatide doses were more efficacious than all comparators with respect to BW loss. Any of the three doses was superior to GLP-1 RAs in achieving significant weight-loss (Table 3). The superiority of tirzepatide with respect to BW control was more effective in the comparison versus insulin (Table 3). Furthermore, our system-review results have showed evidence of a reduction in bodyweight with subjects dosed with all tirzepatide doses compared with all comparators in the all eligible RCTs. Five clinical trials of SURPASS 1–5 in T2DM subjects have shown that Tirzepatide at 5, 10 or 15 mg weekly reduces BW (5.4 to 11.7 kg) by amounts unprecedented for a single agent [16–18, 21, 22].

#### Lipid metabolism

There are two RCTs were included to compare the changing in lipid metabolism between the tirzepatide and placebo/control groups. As 40/26 weeks follow-up, the lipid metabolism was significantly improved in all tirzepatide dose treatment groups. More data details are summarized in Table 2. For Triglycerides (TG), the results of present meta-analysis indicated that there was a significant reduction in all three tirzepatide doses groups with a dose-dependent reduction (SMD = − 5.651, 95% CI: − 5.816 to − 5.485, P < 0.05). However, the opposite results were obtained for Lipoprotein cholesterol (LDL-c). Meta-analysis results show that tirzepatide is not as effective as the control group in reducing LDL-c excluding 5 mg subgroup. For HDL cholesterol (HDL-c) level, there was a substantially increased following tirzepatide administration compared with the placebo/control group (SMD = 2.974, 95% CI: 2.869 to 3.079, P < 0.05). Of note, the type of anti-hyperglycaemic agents and mean dose during the study duration were 1 mg/week with semaglutide [22], and 1.5 mg/week with dulaglutide, respectively, in the two trials [23]. Due to the insufficient number of trials reporting lipid outcomes, no analysis was made on other lipid species (such as Total cholesterol).

### Safety and tolerability of tirzepatide

#### All adverse events

**Fig. 4:**
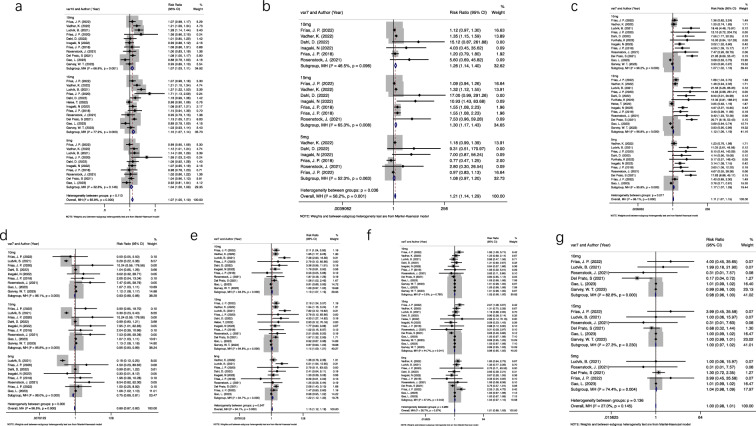
Summary of count data analysis with random effects or fixed effects ORs (95% CI) estimate with a p-value for analysis of primary safety outcomes. *Statistically significant variables at P value < 0.05. **a** All adverse events, **b** gastrointestinal events, **c** decreased appetite, **d** hypoglycemic events, **e** discontinuation of therapy, **f** serious adverse events, **g** fatal adverse event

Compared with Insulin, AEs were more frequent with all tirzepatide doses, especially 15 mg (RR = 1.12; 95% CI 1.06 to 1.18) (Table 4, Fig. 4). Compared with GLP-1 Ras, more subjects receiving Tirzepatide 10 mg and 15 mg experienced AEs, while no statistical significance was observed for Tirzepatide 5 mg (Table 4). Of note, there was no statistical significance be found when comparing all doses of tirzepatide with placebo separately (P ≥ 0.05, Table 5). These AEs were more common in the 10 mg and 15 mg cohorts compared with lower (5 mg) cohort, indicating a dose-dependent increase in AEs production correlating with increased Tirzepatide.

**Table 4:** Summary of count data analysis with Random effects or fixed effects ORs (95% CI) estimate with a p-value for analysis of primary safety outcomes

Primary safety outcomes	5 mg	10 mg	15 mg	Overall
All adverse events	1.04 (1.00, 1.09) P ≥ 0.05	1.07 (1.03, 1.11) P < 0.05	1.10 (1.07, 1.14) P < 0.05	1.07 (1.05, 1.10) P < 0.05
Serious adverse events	1.04 (0.97, 1.10) P ≥ 0.05	1.02 (0.98, 1.05) P ≥ 0.05	0.99 (0.95, 1.02) P ≥ 0.05	1.01 (0.99, 1.03) P ≥ 0.05
Hypoglycemic events	0.75 (0.69, 0.81) P < 0.05	0.93 (0.89, 0.98) P < 0.05	0.75 (0.69, 0.81) P < 0.05	0.89 (0.87, 0.92) P < 0.05
Gastrointestinal events	1.078 (0.965, 1.203) P ≥ 0.05	1.262 (1.138, 1.400) P < 0.05	1.296 (1.172, 1.432) P < 0.05	1.213 (1.142, 1.289) P < 0.05
Discontinuation of therapy	1.22 (1.12, 1.33) P < 0.05	1.13 (1.07, 1.19) P < 0.05	1.15 (1.09, 1.21) P < 0.05	1.15 (1.12, 1.19) P < 0.05
Fatal adverse events	1.04 (0.98, 1.09) P ≥ 0.05	0.98 (0.96, 1.00) P ≥ 0.05	1.00 (0.97, 1.02) P ≥ 0.05	1.00 (0.98, 1.01) P ≥ 0.05
Decreased appetite	3.213 (1.423, 7.256) P < 0.05	2.963 (1.153, 7.612) P < 0.05	1.12 (1.06, 1.19) P < 0.05	3.021 (1.863, 4.901) P < 0.05

*Statistically significant variables at P value < 0.05

**Table 5:** Summary of count data analysis with random effects or fixed effects ORs (95% CI) estimate with a p-value for analysis of primary safety outcomes based on various type of comparators

Primary safety outcomes	Comparator	5 mg	10 mg	15 mg
All adverse events (AEs)	GLP-1 RA	1.05 (0.99, 1.11) P ≥ 0.05	1.11 (1.05, 1.17) P < 0.05	1.12 (1.06, 1.18) P < 0.05
	Insulin	1.203 (0.97, 1.09) P ≥ 0.05	1.06 (1.01, 1.12) P < 0.05	1.12 (1.06, 1.18) P < 0.05
	Placebo	1.10 (0.94, 1.30) P ≥ 0.05	1.00 (0.92, 1.10) P ≥ 0.05	1.04 (0.95, 1.13) P ≥ 0.05
	overall	1.04 (1.00, 1.09) P ≥ 0.05	1.07 (1.03, 1.11) P < 0.05	1.10 (1.07, 1.14) P < 0.05
Serious adverse event	GLP-1 RA	1.54 (1.08, 2.19) P < 0.05	1.30 (0.90, 1.88) P ≥ 0.05	1.32 (0.91, 1.89) P ≥ 0.05
	Insulin	0.99 (0.93, 1.05) P ≥ 0.05	1.00 (0.94, 1.06) P ≥ 0.05	0.96 (0.91, 1.02) P ≥ 0.05
	Placebo	1.86 (0.47, 7.31) P ≥ 0.05	1.01 (0.98, 1.04) P ≥ 0.05	0.98 (0.95, 1.01) P ≥ 0.05
	overall	1.04 (0.97, 1.10) P ≥ 0.05	1.02 (0.98, 1.05) P ≥ 0.05	0.99 (0.95, 1.02) P ≥ 0.05
Hypoglycemic events	GLP-1 RA	1.39 (0.45, 4.31) P ≥ 0.05	1.85 (0.63, 5.42) P ≥ 0.05	1.39 (0.45, 4.31) P ≥ 0.05
	Insulin	0.72 (0.67, 0.78) P < 0.05	0.78 (0.072, 0.84) P < 0.05	0.72 (0.67, 0.78) P < 0.05
	Placebo	8.37 (1.07, 65.51) P < 0.05	1.16 (1.11, 1.22) P < 0.05	8.37 (1.07, 65.51) P < 0.05
	overall	0.75 (0.69, 0.81) P < 0.05	0.93 (0.89, 0.98) P < 0.05	0.75 (0.69, 0.81) P < 0.05
Gastrointestinal events	GLP-1 RA	1.062 (0.951, 1.186) P ≥ 0.05	1.231 (1.110, 1.366) P < 0.05	1.260 (1.140, 1.392) P < 0.05
	Insulin	–	–	–
	Placebo	2.802 (0.296, 26.544) P ≥ 0.05	5.603 (0.685, 45.822) P ≥ 0.05	7.533 (0.957, 59.279) P ≥ 0.05
	overall	1.078 (0.965, 1.203) P ≥ 0.05	1.262 (1.138, 1.400) P < 0.05	1.296 (1.172, 1.432) P < 0.05
Discontinuation of therapy	GLP-1 RA	1.34 (0.94, 1.91) P ≥ 0.05	1.92 (1.38, 2.67) P < 0.05	1.95 (1.41, 2.71) P < 0.05
	Insulin	1.19 (0.11, 1.28) P ≥ 0.05	1.12 (1.03, 1.22) P < 0.05	1.19 (1.10, 1.29) P < 0.05
	Placebo	1.55 (0.38, 6.38) P ≥ 0.05	1.01 (0.98, 1.05) P ≥ 0.05	0.98 (0.94, 1.03) P ≥ 0.05
	overall	1.22 (1.12, 1.33) P < 0.05	1.13 (1.07, 1.19) P < 0.05	1.15 (1.09, 1.21) P < 0.05
Fatal adverse events	GLP-1 RA			
	Insulin	1.03 (0.98, 1.08) P ≥ 0.05	0.95 (0.91, 1.00) P ≥ 0.05	0.98 (0.93, 1.03) P ≥ 0.05
	Placebo		0.98 (0.96, 1.00) P ≥ 0.05	1.00 (0.99,1.00) P ≥ 0.05
	overall	1.04 (0.98, 1.09) P ≥ 0.05	0.98 (0.96, 1.00) P ≥ 0.05	1.00 (0.97, 1.02) P ≥ 0.05
Decreased appetite	GLP-1 RA	1.62 (1.20, 2.19) P < 0.05	1.62 (1.20, 2.19) P < 0.05	1.64 (1.31, 2.06 P < 0.05
	Insulin	1.02 (0.94, 1.10) P ≥ 0.05	1.05 (0.96, 1.15) P ≥ 0.05	1.09 (1.00, 1.19) P ≥ 0.05
	Placebo	12.68 (1.63, 98.56) P < 0.05	0.99 (0.94, 1.03) P ≥ 0.05	1.00 (0.96, 1.05) P ≥ 0.05
	overall	1.17 (1.07, 1.29) P < 0.05	1.07 (1.02, 1.13) P < 0.05	1.12 (1.06, 1.19) P < 0.05

*Statistically significant variables at P value < 0.05

#### Gastrointestinal and decreased appetite events

In clinical trials of tirzepatide intake, the frequently and significantly observed AEs were related to the gastrointestinal system, generally mild or moderate in nature, and nausea, vomiting and diarrhoea were the most commonly adverse effects. Frequency of serious GEs was similar between Tirzepatide and placebo arms. In terms of insulin, statistical analysis was not performed due to limited subgroup. Compared with GLP-1 Ras, gastrointestinal adverse was more frequent with tirzepatide 10 mg and 15 mg, and occurred at a similar incidence on tirzepatide 5 mg (Table 4). However, in comparison with placebo/control group, GEs occurred at a higher incidence on tirzepatide 15 mg.

Decreased appetite was the second most commonly AEs with the incidence was reported ranging from 3.8% to 18.9% in tirzepatide treatment groups [24]. Our meta-analysis results have indicated that all three tirzepatide doses experienced reduced appetite more frequently than all comparators and display some dose-dependency, except Tirzepatide 5 mg. Therefore, it is speculated that tirzepatide produced a more significant effect on glycemic control and weight loss by inhibiting appetite and food intake [11].

#### Hypoglycemic events

Frequency of serious HEs was observed no statistical significance between all tirzepatide dose and GLP-1 Ras arms (Tables 4, 5). However, the incidence of all tiracetide doses (5, 10 and 15 mg) was significantly lower than Insulin in terms of hypoglycemia. Compared with Placebo, more subjects taking Tirzepatide experienced hypoglycaemia (Table 4). Evaluation of the total hypoglycemic events across all included articles yielded a significant decrease in HEs when all dose groups compared to the control group (Tables 4, 5).

#### discontinuation of therapy

Our current results found that DT caused by AEs was similar between any of the tirzepatide doses and placebo arms. Compared with GLP-1 Ras, more subjects receiving Tirzepatide 10 mg and 15 mg experienced DT, with no significant difference for Tirzepatide 5 mg (Table 4). Compared with insulin, DT due to adverse events occurred at a higher incidence on Tirzepatide 10 mg and 15 mg dose. Therefore, Tirzepatide increased odds of DT when compare with GLP-1 RAs or insulin.

#### Serious and fatal adverse events

Individual serious AEs have been found no difference in either arm, except Tirzepatide 5 mg compared to GLP-1 Ras. (Tables 4, 5). Across all trials, none of the deaths were considered by the investigators to be related to tirzepatide. We speculate that Tirzepatide was not associated with increased rates of serious AEs and all-cause mortality.

Specifically, participants treated with weekly tirzepatide achieved HbA1c and BW target values significantly lower than any other comparator without clinically significant increase in the incidence of hypoglycemic events, serious and all-cause fatal adverse events when compared with placebo/controls. However, gastrointestinal adverse events and decreased appetite events were reported more frequently with tirzepatide treatment than with placebo/controls.

## Discussion

This systematic review and meta-analysis was designed to summary and synthesis the main efficacy and safety outcomes from the most up-to-date RCTs of weekly tirzepatide doses of 5 mg, 10 mg and 15 mg in individuals with type 2 diabetes. Based on our meta-analysis results, the dose-dependent reduction of HbA1c, FBG and BW induced by tirzepatide compared with placebo and weekly GLP-1 receptor antagonist, as well as insulin regimen has important clinical significance. In terms of lowering of lipid, Tirzepatide resulted in a dose-dependent improve of TG when compared with the GLP-1 Ras (1 mg/week with semaglutide and 1.5 mg/week with dulaglutide). Moreover, our system-review found the most commonly observed side effects were GE and decreased appetite in comparison with various anti-hyperglycaemic agents. The decreased appetite might be contributed to the reduction in weight and it is consistent with previous research reports [25], indicating a more profound effective on regulating food intake and satiety compared with GLP-1 RAs. [26] Considering the gastrointestinal system, study has suggested the lower incidence of treatment-related gastrointestinal system did seem to be associated with Lower initial dose and smaller subsequent dose increment [27]. The incidence of GE and discontinuation due to AEs were similar when compared Tirzepatide with placebo. Therefore, we speculated that the increased odds of DT vs all comparators might be attributed to the severity of GEs experienced with tirzepatide. Notably, this beneficial hypoglycemic effect of tirzepatide was not associated with increased incidence of hypoglycemic events, serious and all-cause fatal adverse events. These results indicate it is possible to achieve well established, but stringent, individualizing the glycemic and BW targets for diabesity patients in a safe manner.

We identified three previous systematic review and meta-analysis with tirzepatide treatment for T2DM, which included four (2783 participants), seven (6609 participants) and six trials (3484 participants), respectively, thorough literature retrieve and review [28–30]. Discrepancies of include literatures and research methodology render the results of these meta-analysis non-comparable to our findings. Specifically, Bhagavathula et al. summarized the efficacy results in meta-analysis, regardless of the type of comparators (insulin, GLP-1 Ras or placebo) [28]. In the meta-analysis of Thomas Karagiannis and colleagues [29], no analysis was performed on the efficacy of blood glucose and blood lipid. The three articles have important limitation of insufficient literature. Dutta, D and colleagues [30] did not analysis base on therapeutic doses and comparators. Given the availability of new outcomes data and relative importance of this study, we have updated this previous literature search and meta-analysis. Instead, we opted to summary and synthesis systematic review results and produce meta-analysis estimates with potential clinical relevance and value by conducting separate analysis based on different therapeutic doses (5 mg, 10 mg and 15 mg) and type of comparators for HbA_1c_, FBG, BW and lipid outcomes with included all RCTs of tirzepatide in the treatment of T2DM published to date. Moreover, the present meta-analysis provides a comprehensive assessment of safety and tolerability results, which is also important the evaluation of efficacy when opting an optimal therapy in clinical treatment.

Recently, unimolecular, multifunctional peptides combining GLP-1Ra with GIP has been considered as a promising therapeutic agent for insight against T2DM, suggested that these two incretins can act on β-cells through distinct metabolic effects synergistically and complementarily [31]. Acting on both GIP and GLP-1 receptors to potentiate glucose-induced insulin secretion and improve glucose tolerance is attractive because the combination of these mechanisms is hypothesized that the metabolic action of GIP adds to the established clinical benefits of selective GLP-1 Ras, decreasing energy consumption, improving white adipose tissue health and function, increasing insulin response and glucagonostatic response [11]. Studies have shown that the GIP/GLP-1Ras action did not affect the incretin effects on GIP-stimulated insulin secretion, and strengthened the inherent efficacy and broadened their therapeutic range when both the GIP receptor and GLP-1 receptor are activated [32, 33]. We reviewed the multiple functions of GIP/GLP-1RAs in regulating metabolism and energy balance in the contexts of up-to-date findings in T2D indicating that dual GIP/GLP-1As therapy produced profound weight loss, glycemic and BFG control, and lipid improving.

Specifically, multi-functional peptides of GLP-1RAs improve glycemic control by stimulating the glucose-induced insulin secretion [34, 35], delaying gastric empty [36, 37], and limiting plasma glucagon level [38], and activating anorexigenic pathways to inhibit of appetite and food intake [39]. The main difference between GIP and GLP-1Ras is stimulation of plasma glucagon release. Unlike GLP-1, GIP is reportedly glucagonotropic in normoal and/or hypoglycemic state, and normally suppresses glucagon secretion in the hyperglycaemic state [40]. Regarding adipose tissue regulation of GIP, the consistent report has yet to be established. Some studies had investigated the biological activity of GIP on adipocytes and indicated that GIP is implicated in adipose tissue mass and metabolism by regulating glucose uptake [41], lipolysis [42], and the activity of lipoprotein lipases [43, 44], some of which suggest the adipogenic effect of GIP with studies indicating GIPR knockout mice and chronic elevation of serum GIP levels in a transgenic mouse would inhabit diet-induced obesity [44, 45]. Furthermore, other studies demonstrated that acute GIP infusion to human would increase adipose tissue blood flow [46], promote insulin sensitivity, glucose tolerance and β-cell function [45]. A hyperglycemic clamp study has found that GLP-1R expression decreased but GIP R expression increased under the effect of acute hyperglycemia, and is similar with the experimental results of culturing at high-glucose concentrations for 48 hours [47]. Peptide engineering enables the synthesizing of structural motifs, which would be a hybrid peptide with dual agonism [48, 49]. The GLP-1 Ras acts synergistically with GIP activation gain a broad improvement in metabolic health with the hypothesis that enhancing insulin secretion by dual actions on pancreatic β cells [50], allowing greater weight loss [11], improving glycemia [29], restoring sensitivity to GIP [51] and additional mechanisms of actions [5, 11] when compared with single GLP-1 RA or GIP. Therefore, combining the GLP-1 Ras with GIP receptors would produce an effective treatment for diabetes with optimum individualizing the glycemic and BW targets.

Terzepatide is currently the first hybrid peptide with dual GIP/GLP-1 receptor co-agonist approved for improving glycaemic control in patients with T2D, as an adjunct to diet and exercise, in the United States, Europe and the United Arab Emirates [24]. In addition to the above efficacy, Tirzepatide has been demonstrated to improve intrahepatic triglycerides in T2D when compared to insulin degludec [52], which may provide new therapeutic strategy for patients with fatty liver as describing for GLP-1 RAs [53]. Although this compound with subcutaneous injection weekly have achieved unprecedented results in glucose control and weight loss employed in many clinical trials, the quantities may significantly change the potential pathogenesis of T2D [54] and is related to the remission of diabetes [55, 56]. In addition, there are some novel questions with respect to pathogenesis should be addressed as the physiological changing of diabetes, such as disease progression, long-term prognosis, macro- and microvascular complications. More clinical practices are warranted to further integrate long-term efficacy, safety and cost-effectiveness with country-specific cost-utility analysis comparing tirzepatide with various anti-hyperglycaemic agents or independent of this, based on health technology assessments.

At present, more compounds of receptors with dual agonists are being tested, for example, GLP-1R/GR (glucagon receptor), GLP-1R/AR (amylin receptor) and GLP-1R/NPYR (peptide YY binds to neuropeptide Y receptors) [57, 58], and might achieve further advances in T2DM, obesity and associated conditions therapy.

### Limitation

Unavoidably, there are several potential limitations in this meta‐analysis. First, the demographics and clinical characteristics of participants often differ (such as background glucose-lowering treatment, Concomitant medication, and intervention duration). Second, the open-label design should be considered as susceptible to subjectivity. Thrid, using self-report assessment for gastrointestinal adverse events has limitations [59]. An increased risk of nocebo effect and/or placebo effect should be considered to assess AEs [60]. Final, other residual confounding factors effected on glycaemic control, such as patients with asymptomatic gastroparesis, could not be excluded either. Based on the mentioned above, those confusions may be the main source of this study limitations.

## Conclusion

The dual GIP/GLP-1 receptor co-agonist, tirzepatide, for diabetes therapy has opened a new era on personalized glycemia control and weight loss in a safe manner with broad and promising clinical implications. Specifically, we reviewed the multiple functions of GIP/GLP-1RAs in regulating metabolism and energy balance in the context of up-to-date findings in T2DM indicating that dual GIP/GLP-1As therapy produced profound weight loss, glycemic and BFG control, and lipid lowering. The results of this systematic review and meta-analysis indicate it is possible to achieve well established, but stringent, individualizing the glycemic and BW targets for diabesity patients in a safe manner. More clinical practices are warranted to further integrate long-term efficacy, safety and cost-effectiveness with country-specific cost-utility analysis comparing tirzepatide with various anti-hyperglycaemic agents or independent of this, based on health technology assessments.

### Supplementary Information

**Additional file 1: Table S1.** PRISMA 2020 item checklist for abstracts and reporting guideline.

## Data Availability

On request, data were extracted from original research and data used in meta-analyses are accessible.
